# A 43-year-old woman on triptorelin presenting with pseudotumor cerebri: a case report

**DOI:** 10.1186/1752-1947-6-122

**Published:** 2012-05-03

**Authors:** Uday Kumar Bhatt, Imran Haq, Venkata S Avadhanam, Kim Bibby

**Affiliations:** 1 Department of Ophthalmology, Queen’s Medical Centre, Nottingham, NG7 2UH, UK; 2 Birmingham and Midland Eye Centre, Dudley Road, West Midlands, B18 7QH, UK; 3 Sussex Eye Hospital, Brighton, East Sussex, BN2 5BF, UK; 4 Department of Ophthalmology, Leicester Royal Infirmary, Leicester, LE1 5WW, UK

## Abstract

**Introduction:**

To the best of our knowledge, this is the first time triptorelin has been reported to cause benign intracranial hypertension.

**Case presentation:**

A 43-year-old Caucasian woman who suffered from chronic menorrhagia was started on triptorelin, a gonadotrophin-releasing hormone analogue. Three days later, she developed gradually worsening headaches accompanied by bilateral visual disturbance. Examination revealed bilateral papilledema and enlarged blind spots on her visual fields. A diagnosis of benign intracranial hypertension was made and confirmed on magnetic resonance imaging.

**Conclusion:**

We recommend that patients at high risk (women who are overweight and of reproductive age) who are using any gonadotrophin-releasing hormone analogue (for example, triptorelin) should be periodically monitored for the possible development of benign intracranial hypertension.

## Introduction

A woman with menorrhagia was started on triptorelin, a synthetic gonadotrophin-releasing hormone analogue, mainly used in the treatment of prostate carcinoma and endometriosis.

Three days later, she developed gradually worsening headaches accompanied by bilateral visual disturbance. An examination revealed bilateral papilledema and enlarged blind spots on her visual fields. A diagnosis of benign intracranial hypertension (BIH) was made and confirmed on magnetic resonance imaging (MRI).

## Case presentation

A 43-year-old Caucasian woman was treated for chronic menorrhagia with a Mirena® coil and traxenamic acid with no success. Her gynecologist started her on triptorelin. Three days after the first dose, she attended our eye centre casualty with progressively worsening headaches accompanied by visual disturbances. She had no past medical history of note, and was on no other medications at the time of presentation.

**Figure 1:**
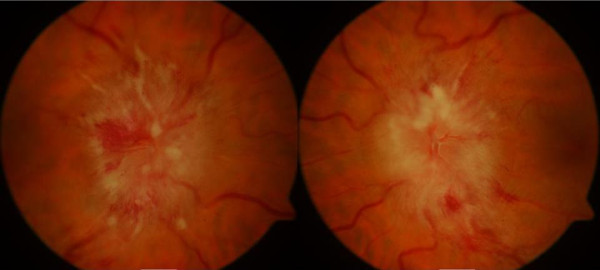
Fundal photographs showing bilateral papilledema.

**Figure 2:**
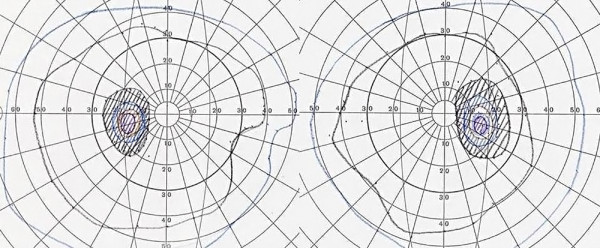
Goldmann visual fields displaying bilateral enlarged blind spots.

**Figure 3:**
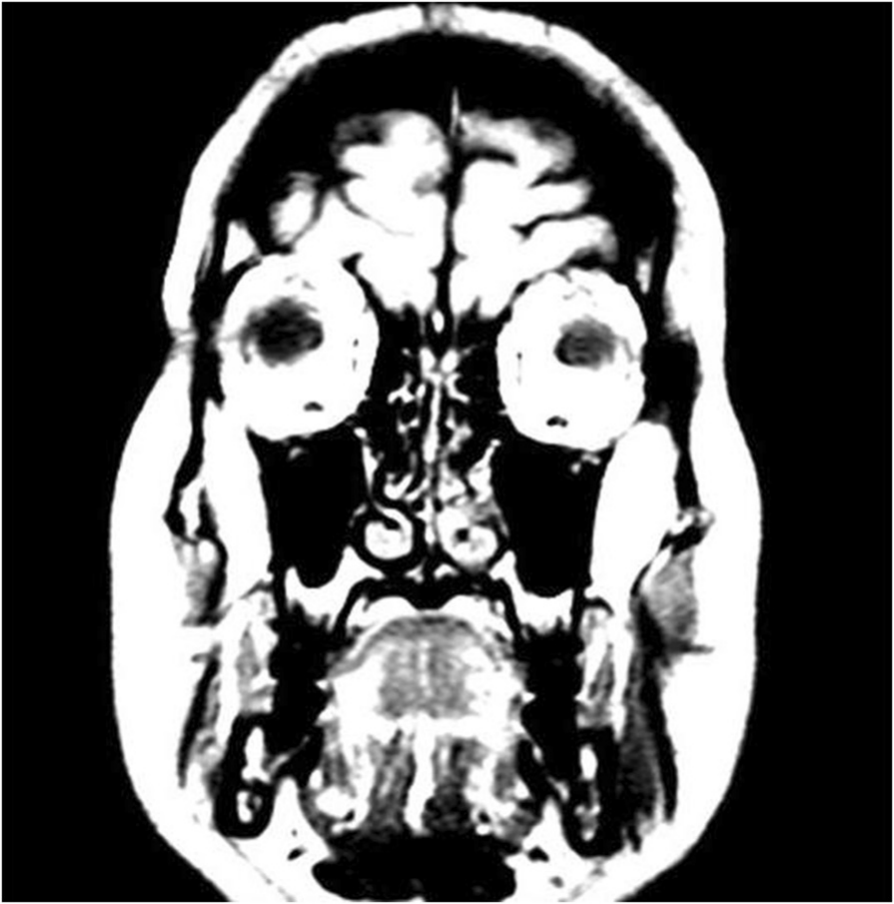
Magnetic resonance imaging displaying capacious cerebral spinal fluid spaces around the optic nerves.

She had unaided Snellen acuity of 6/36 improving to 6/18 with pinhole in either eye. Her color vision, pupils and anterior segments were normal but she had bilateral papilledema (Figure 1) and enlarged blind spots on her visual fields (Figure 2). She was overweight (not obese) with a body mass index of 28. She did not report any recent-onset weight gain. The rest of her physical examination was unremarkable. MRI showed no intracranial mass or ventricular dilatation but she had capacious cerebral spinal fluid (CSF) spaces around her optic nerves (Figure 3). A simultaneous magnetic resonance venogram (MRV) displayed normal cerebral venous sinuses.

She was urgently referred to our neurology team for a lumbar puncture. Her CSF opening pressure was significantly high at 43 cm but a CSF analysis was normal. A diagnosis of BIH was made and she had further therapeutic CSF drainage done at the same time. She was advised to discontinue triptorelin and start oral acetazolamide 250 mg four times a day, although this was only given for seven days and stopped, as our patient could not tolerate it.

**Figure 4:**
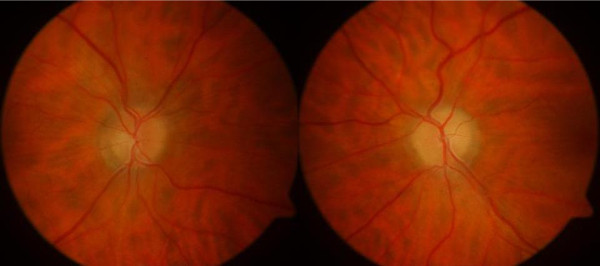
Resolution of papilledema.

Within the next three days, her headaches ceased and her visual acuity began improving gradually over the next three weeks to 6/5 in both eyes. At the same time, the papilledema settled with near normal appearance of the discs (Figure 4). A month later, she had a normal CSF opening pressure on a lumbar puncture. Her visual fields showed remarkable improvement with normalization of the blind spots over the next three months.

## Discussion

BIH, also known as pseudotumor cerebri, has an incidence of 1.6 to 3.5 per 100,000 in women and is much higher at 7.9 to 20 per 100,000 in women who are overweight and of reproductive age [1]. Although by definition the etiology of BIH is unknown, a plethora of drugs and systemic conditions are associated with this condition [2,3]. To explain the mechanism of BIH, some hypotheses have been put forward. These include the link between BIH and relatively obstructive segments in the distal transverse sinus, unrecognized sinus thrombosis (undetected by MRI or MRV scans), or the presence of increased arterial inflow with an accompanying low-grade stenosis of the transverse sinus [2-5].

Administration of triptorelin initially stimulates the anterior pituitary gland to release the gonadotrophins luteinizing hormone and follicle-stimulating hormone. However, its sustained use leads to their suppression.

To explain the BIH in this case, we speculate that the Triptorelin-induced initial surge of gonadal steroids may have caused non-occlusive thrombosis of the dural venous sinuses by creating a venous hypertensive state and impeding CSF drainage.

BIH usually develops several weeks to months after the intake of the offending medication [6]. In our case, the dramatic presentation and the very high CSF opening pressure (43 cm) suggest a possible idiosyncratic effect in our patient. Nevertheless, after stopping the drug, our patient’s visual improvement and the resolution of the papilledema were also very dramatic. Such a causal relationship strongly suggests triptorelin to be the cause of the BIH.

BIH has also been documented with the use of leuprorelin, which is similar in structure to triptorelin, and this only strengthens the basis of our report [7]. In 1994, Radhakrishnan *et al*. [8] reviewed the literature on BIH associated with other diseases and with drugs. Within their study, the authors insisted that the following criteria should be met to include the disease or drug within their list of causally related associations: at least two cases should have been described; the reported cases should have met all the criteria for the diagnosis of idiopathic intracranial hypertension; and intracranial dural sinus thrombosis should have been ruled out with reasonable certainty.

Therefore gonadotrophin-releasing hormone analogues fulfill the authors’ criteria as a causative agent for BIH [8].

## Conclusion

We recommend that patients at high risk (women who are overweight and of reproductive age) who are using any gonadotrophin-releasing hormone analogue (for example, triptorelin) should be periodically monitored for the possible development of BIH.

## Consent

Written informed consent was obtained from the patient for publication of this manuscript and any accompanying images. A copy of the written consent is available for review by the Editor-in-Chief of this journal.

## Competing interests

The authors declare that they have no competing interests.

## Authors’ contributions

UB collected the patient details and was a major contributor in writing the paper. IH helped analyze the paper and was a major contributor in writing the paper. VA and KB reviewed the paper and suggested changes. All authors read and approved the final manuscript.
